# Detoxome Capacity of the Adult Rumen Fluke *Calicophoron daubneyi* Extends into Its Secreted Extracellular Vesicles

**DOI:** 10.1021/acs.jproteome.4c00615

**Published:** 2025-01-20

**Authors:** Nathan Rhys Allen, Kathryn M. Huson, Lukas Prchal, Mark W. Robinson, Peter M. Brophy, Russell M. Morphew

**Affiliations:** † Department of Life Sciences, Aberystwyth University, Aberystwyth, Wales SY23 3DA, U.K.; ‡ School of Biological Sciences, Queen’s University Belfast, Belfast, Northern Ireland BT9 5BY, U.K.

**Keywords:** anthelmintic, detoxome, extracellular vesicle, glutathione transferase, Helminth, rumen fluke

## Abstract

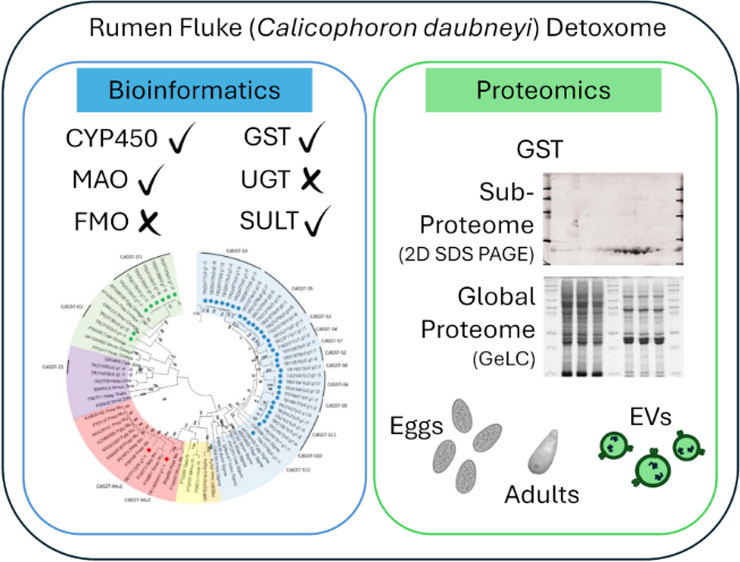

Helminth parasites have long adapted to survive hostile host environments and can likely adapt against the chemical anthelmintic challenge. One proposed adaptation route is via Phase I and II xenobiotic metabolizing enzymes (XMEs). For successful Helminth pharmacotherapy discovery programs, a working understanding of Helminth-derived chemical detoxification, the Helminth detoxome, is a must. At present, the detoxome of a newly emerging Helminth parasite, the rumen fluke *Calicophoron daubneyi*, remains unexplored. Thus, a combined bioinformatics, sub-, and global-proteomic approach has been employed to examine the detoxome of adult *C. daubneyi*. Transcriptome analysis revealed a complement of Phase I (cytochrome P450s and monoamine oxygenases) and Phase II (glutathione transferases [GSTs] and sulfotransferases) XMEs. Affinity-led subproteomic exploration of the GSTs revealed six GST isoforms in adult rumen fluke (CdGST-Mu1-2, S1, and S3–5), with global approaches identifying additional GSTs (CdGST-O1-2, Z1, and S2) and a unique egg-specific variant (CdGST-S6). Examination of *C. daubneyi* extracellular vesicles revealed a GST profile replicating that of the adult with the absence of two isoforms (CdGST-S2 and S4), with an additional identification of a sulfotransferase. These data represent the first exploration into the complete rumen fluke detoxification capacity and will provide direction for future anthelmintic discovery programs.

## Introduction

Parasitic helminths have evolved an array of mechanisms, including some likely undiscovered, facilitating their survival against the effects of anthelmintics.^1^ To date, several classical mechanisms involved in the development of anthelmintic resistance have been identified in Helminth species, such as target site changes and drug metabolism/efflux pathway alterations.^2−4^ Altered metabolism, via inducible Phase I and II xenobiotic metabolizing enzymes (XMEs), is a well-recognized anthelmintic survival mechanism in parasitic helminths, which when additionally coupled to Phase III compound transporters can facilitate the development of resistance to anthelmintics.^1,2^ Therefore, since a key component of absorption, distribution, metabolism, and excretion (ADME) pharmacokinetics is the understanding of XME capacity within the host, an understanding of a parasite's XME repertoire is surely a prerequisite to successful anthelmintic discovery. New parasite XME knowledge will prevent parasite-led anthelmintic neutralization or, in contrast, allow the selective activation of a compound to target parasitic XMEs.

Xenobiotic reduction, hydrolysis, and conjugation reactions in helminths have long been detected via biochemical assays in somatic extracts, acknowledging the role of these Phase I and Phase II enzymes in protecting the organism from the negative effects of these external compounds.^2,5,6^ While XME capacity has been studied in several Helminth species at gene and proteome levels,^6−9^ many other helminths of human and veterinary importance still have not been subject to such in-depth analyses. Furthermore, with anthelmintic-resistant parasitic helminths of livestock widespread, it is important to understand species-specific detoxification mechanisms in order to allow effective future pharmacotherapy,^10^ and XME studies may uncover novel biological pathways to target.^11^ The parasitic rumen fluke *Calicophoron daubneyi*, the causative agent of paramphistomosis in sheep, cattle, and goats, has been increasingly reported across Europe in recent times^12^ and is yet to undergo similar analysis of XME capacity to support future control.

Importantly, the most recent evidence within the UK and EU points to an increased prevalence of paramphistomosis, rumen fluke infection, compared to that of fasciolosis.^12^ Despite this, clinical paramphistomosis and mortality are currently of low incidence throughout the UK and EU. Nevertheless, outbreaks leading to fatalities, in both sheep and cattle, have been documented.^13^ At present, it seems that the newly excysted juvenile (NEJ)/immature rumen fluke is responsible for the majority of clinical signs observed in clinical cases, with the impact of adult fluke residing in the rumen still undefined.^13^ However, adult rumen flukes can still be present in high numbers in livestock, and a large number of adults will lead to the production of a significant number of eggs. Significant numbers of eggs then passed into the environment will underpin future infections and subsequent clinical cases from NEJ infections.

The initial molecular investigations into *C. daubneyi* aimed to generate tools for supporting future functional genomic analysis. In doing so, the first transcriptome for the adult life stage was published supporting some preliminary proteomic investigations of the excretory-secretory products and the somatic fractions.^14^ More recently, transcriptomes of additional intramammalian life stages, together with secretome data sets for NEJ and adult stages, have also been generated,^15^ expanding the tools available to explore *C. daubneyi*. It is now time to put these tools to work to undertake focused functional genomics studies, with our focus on the XME capacity in *C. daubneyi* adults.

With the publication of the first transcriptome of *C. daubneyi*, an initial study into the Phase II detoxification glutathione transferases (GSTs) as key flatworm XMEs was performed.^14^ GSTs are likely the major detoxification enzymes in adult helminths. Their importance is emphasized by an apparent lack of, or reduced expression of, the major Phase I cytochrome P450 (CYP)-dependent detoxification system.^10,16^ For example, in the highly pathogenic flatworm *Fasciola hepatica*, as much as 4% of the total soluble protein in adults is accounted for by GSTs^6^ which are expressed widely in the parasite’s tissue. Furthermore, following in vitro maintenance, *F. hepatica* GSTs are also released in excretory/secretory products, with this widespread abundance indicating important physiological roles,^17,18^ which may also be reflected in the flatworm *C. daubneyi*. Thus, research into *C. daubneyi* GSTs would significantly benefit from further analyses such as those documented for the fasciolids *F. hepatica*(7,17) and *F. gigantica*.^9^ Furthermore, in-depth analysis could support the elucidation of the role GST families play in any anthelmintic response such as that demonstrated for *F. hepatica* under triclabendazole (TCBZ) exposure, highlighting binding to Mu class GSTs.^19^

More recent molecular studies of *C. daubneyi* have identified and characterized the secreted extracellular vesicles (EVs) while demonstrating the first evidence of Helminth-derived EVs modulating components of the host microbiome.^20^ Since their discovery in helminths by Marcilla et al.,^21^ Helminth-derived EVs are now the focus of much investigation aiming to characterize their components and identify biological roles for these membrane-bound structures.^18,22−24^ Interestingly, recent evidence has documented EV-anthelmintic interactions, specifically identifying an interaction between *F. hepatica-*derived EVs and TCBZ following exposure of adult flukes to the drug in vitro.^25^

Given the limited anthelmintic options available for the control of *C. daubneyi*, with only the currently unlicensed oxyclozanide available (see Huson et al.^26^ for the anthelmintics assessed for rumen fluke), and the continuing reports of clinical outbreaks and spread of livestock infection within the EU and UK, it is important to uncover the repertoire of XME capacities within *C. daubneyi* to support and encourage future anthelmintic development. Therefore, this study aimed to reveal the full detoxome of adult *C. daubneyi* using a combined bioinformatics and proteomics research platform.

## Materials and Methods

### Bioinformatic Identification of Potential Detoxome Components

Sequences encoding known detoxification proteins, namely, GSTs, cytochrome P450 (CYPs), sulfotransferases (SULTs), flavin-containing monooxygenases (FMOs), monoamine oxygenases (MAOs), and UDP-glucurosyltransferase (UDP), from model organisms and genome sequenced helminths were retrieved from the GenBank database (http://www.ncbi.nlm.nih.gov/; full details of the sequences used are available in the Supporting Information). The adult *C. daubneyi* transcriptome (accession number: GFUT01000000; available at https://sequenceserver.ibers.aber.ac.uk/(27)) was interrogated via a local tBLASTn analysis using the query sequences with a significant similarity cutoff set at 1.00 × 10^–10^. Retrieved sequences were translated (Expasy Translate; https://web.expasy.org/translate/), and the resulting protein sequences were subject to BlastP against the NCBInr database.^28^ All retrieved sequences were then subjected to Pfam^29^ and Interpro^30^ searches in order to identify conserved functional domains from characterized detoxification proteins. Following retrieval of proteins from known detoxification families, sequences retrieved from the transcriptome and representative sequences were uploaded to BioEdit^31^ and subject to ClustalW^32^ multiple sequence alignment analysis, allowing the identification of conserved regions. Multiple sequence alignments were also utilized for phylogenetic analysis using neighbor-joining bootstrap trees produced in MEGA v7.0^33^ with 1000 bootstraps and a Poisson correction allowing visualization of relationships and homology with known classes. Transcript expression levels for individual *C. daubneyi* GST isoforms were analyzed following Huson et al.^15^ Each specific GST isoform was used to tBLASTn the transcriptome to identify the respective expression level expressed as transcripts per million (TPM) values.

### Protein Sample Preparation

#### Sample Collection

Rumen flukes were retrieved from freshly slaughtered bovine rumens at either a local Welsh abattoir (Llanidloes, UK) for adult and egg proteomics or from Northern Ireland (Dungannon, UK) for EV production and proteomics. All flukes were washed in sterile phosphate-buffered saline (PBS) (pH 7.4) at 39 °C to remove contaminating materials. PBS washes were retained for the egg collection. Flukes were then maintained in an RPMI-1640 culture medium containing 0.1% w/v glucose, 100 U/mL penicillin, and 100 μg/mL streptomycin (Sigma-Aldrich), together at a ratio of 1 worm/mL for 5 h at 39 °C. Both flukes and maintenance media were snap-frozen in liquid nitrogen and stored at −80 °C for subsequent somatic and EV GST analyses. Adult flukes were frozen individually. Replicate maintenance media, as batches of 500 mL, were also frozen individually. Maintenance media for EV proteomics was utilized fresh.

#### Somatic Fraction Preparation

Adult samples stored at −80 °C were defrosted on ice and homogenized in batches of 10 in 5 mL of homogenization buffer containing 20 mM potassium phosphate (pH 7.4), 0.1% v/v Triton-X 100, and a mini cOmplete protease inhibitor (Roche, U.K.; added at 1 tablet per 10 mL of buffer). Homogenization was achieved utilizing a glass–glass homogenizer at 4 °C. The homogenate was subjected to centrifugation at 100,000 × *g* for 45 min at 4 °C, and the resulting soluble supernatant was retained. Cytosolic samples were then precipitated using 20% w/v TCA in acetone before being resolubilized in a buffer containing 8 M urea, 2% w/v CHAPS, and 33 mM DTT as detailed by Morphew et al.^34^

#### Soluble Egg Extract Preparation

Eggs were collected fresh from PBS washes carried out on parasites prior to culture. PBS washes were retained and washed through a series of 300, 150, and 45 μm mesh sieves, allowing removal of debris and isolation of eggs. Eggs were collected on the 45 μm sieve and washed with ddH_2_O into a measuring cylinder and left for 10 min to sediment. The resulting supernatant was aspirated and repeated 3 times, allowing further removal of debris. The resulting samples were confirmed as *C. daubneyi* eggs through morphological characteristics utilizing light microscopy.

Isolated eggs were submitted to initial centrifugation at 2000 × *g* at 4 °C, allowing eggs to be pelleted and the supernatant removed. Eggs (approximately 200,000 per replicate) were resolubilized following the method of Moxon et al.^35^ in 2 mL of homogenization buffer (20 mM potassium phosphate pH 7.4, 0.2% v/v Triton-X 100, and cOmplete mini EDTA-free protease inhibitor (Roche, U.K)). Eggs were placed in a mortar and pestle, cooled through the addition of liquid nitrogen, and homogenized. Debris was removed from the sample through centrifugation at 14,000 × *g* at 4 °C for 1 min, and the supernatant containing the soluble protein extract was retained and stored at −20 °C for future analysis.

#### EV Fraction Preparation

Adult *C. daubneyi* EVs were isolated by differential centrifugation as previously described by Cwiklinski et al.^36^ Briefly, after the 5 h incubation period, the fresh parasite maintenance medium was collected and centrifuged at low speed, 300 × *g* for 10 min followed by 700 × *g* for 30 min, to remove large debris. The resulting supernatant was centrifuged at 15,000 × *g* for 45 min at 4 °C to obtain large vesicles, termed 15 K EVs, which were resuspended in 100 μL of PBS. The remaining supernatants were then filtered using a 0.2 μm ultrafiltration membrane and centrifuged at 120,000 × *g* for 1 h at 4 °C to recover smaller vesicles, termed 120 K EVs, that were subsequently washed with PBS and resuspended in 100 μL of PBS. In addition, a second preparation of *C. daubneyi* EVs was purified from frozen culture media following the method of Marcilla et al.^21^ for GST purification. Maintenance media was thawed and initially centrifuged at 300 × *g* for 10 min at 4 °C and then at 700 × *g* for 30 min at 4 °C followed by ultracentrifugation at 100,000 × *g* for 80 min at 4 °C to generate a combined EV preparation. The EV pellet was washed in 5 mL of PBS and agitated until suspended. The sample was again ultracentrifuged at 100,000 × *g* for 80 min at 4 °C, with the resulting pellet resuspended in 200 μL of PBS and stored at −80 °C for future experimentation.

#### GST Purification and Activity Assays

GSTs were purified from the adult somatic, egg, and lysed EV fractions through glutathione (GSH)-affinity chromatography following the method described by Simons and Vander Jagt^37^ as previously described by Chemale et al.^7^ using batches of 10 worms, 200,000 eggs, or EVs purified from 100 worms. Briefly, protein fractions were applied to a glutathione-agarose (GSH agarose, Sigma-Aldrich, U.K.) affinity column. GSTs in samples were purified at 4 °C according to the manufacturer’s instructions. Eluted proteins were concentrated using 10 kDa filters (Amicon Ultra, Millipore) and quantified using the Bradford reagent^38^ as previously described. This process was repeated for all biological replicates. Enzymatic activity was determined spectrophotometrically by assessing the change in absorbance at 340 nm brought about by the conjugation of reduced glutathione (GSH) with 1-chloror-2,4-dinitrobenzene (CDNB). Purified GSTs were assayed for GST activity in 1 mL volumes under conditions detailed according to the method of Habig et al.^39^ (i.e., 1 mM CDNB, 1 mM GSH, pH 6.5, 25 °C). All assays were performed in triplicate with a Cary 50 Bio UV–vis spectrophotometer (Varian, U.K.).

#### Protein Preparation and SDS PAGE

Protein concentrations were determined through Bradford protein estimation (Sigma), following the manufacturer’s protocol.^38^ For 1D SDS PAGE, a Laemmli protein 4 × sample buffer (Biorad) was added to each sample (3:1 ratio) and heated to 95 °C for 10 min. Adult somatic and egg soluble extract samples were then loaded into 7 cm 12.5% Tris/glycine polyacrylamide gels and run using the Protean III system (BioRad). A total of 15 and 120 K EV proteins were separated on reducing 4–12% NuPAGE Bis-Tris gels (Life Technologies). Gels were fixed (40% ethanol (v/v), 10% acetic acid (v/v)) and stained with colloidal Coomassie Brilliant blue (Sigma). Gels were imaged using a GS-800 calibrated densitometer (Biorad).

For two-dimensional SDS PAGE (2DE) protein arrays, GST samples were resuspended in 8 M urea, 2% w/v CHAPS, 33 mM DTT, and 0.5% carrier ampholytes v/v (Biolyte 3/10) and submitted to isoelectric focusing using 7 cm linear pH 3–10 immobilized pH gradient (IPG) strips (BioRad, U.K). IPG strips were rehydrated overnight and focused on a Protean IEF Cell (BioRad) to approximately 10,000 VH. Focused IPG strips were equilibrated for 10 min in a reducing equilibration buffer (30% v/v glycerol, 6 M urea, 1% DTT) followed by 10 min in an alkylating equilibration buffer (30% v/v glycerol, 6 M urea, 4% iodoacetamide). IPG strips were run upon SDS PAGE (12.5% acrylamide) using the Protean II xi 2-D Cell (BioRad). Gels were stained with colloidal Coomassie Brilliant blue (Sigma) and scanned on a GS-800 calibrated densitometer (BioRad).

#### Mass Spectrometry–Tryptic Digestion

For 2DE array protein spots or for the global GeLC proteomic approaches for adults and soluble egg extracts, spots and bands were manually excised from protein gels, destained, and digested overnight with 10 ng/μL sequencing-grade trypsin (Promega) at 37 °C as described by Morphew et al.^40^ For 15 and 120 K EV GeLC approaches, bands were digested with 100 ng/μL sequencing-grade trypsin (Promega) overnight at 37 °C. The digestions were stopped by the addition of trifluoroacetic acid (TFA) to a final concentration of 0.1%. Tryptic peptides were dried in a vacuum centrifuge and reconstituted with 10–20 μL of 0.1% TFA before being analyzed by LC-MS/MS.

#### Mass Spectrometry–LC-MS/MS and Database Searches

Trypsin digested samples for adults and egg soluble extracts were analyzed using a liquid chromatography-tandem mass spectrometer (Agilent 6550 iFunnel Q-Tof) combined with an HPLC-Chip (1200 series, Agilent Technologies, U.K.). Each sample was injected into an enrichment column within the system at a flow rate of 2.5 μL/min using an automated micro sampler with an injection volume of 2 μL in the resuspension buffer 0.1% v/v formic acid and allowed to separate at 300 nL/min. Enrichment and separation were carried out on a Polaris chip (G4240–62030, Agilent Technologies, U.K). A system of solvents was utilized over the process, solvent A (Milli-Q water containing 0.1% formic acid) and solvent B (90% v/v acetonitrile containing 0.1% v/v formic acid). Chromatography was achieved using a linear gradient of 3–8% solvent B over 6 s, 8–35% solvent B over 15 min, 35–90% solvent B over 5 min, and finally 90% solvent B for 2 min.

For trypsin digested samples from 15 and 120 K EV samples, peptides in 5 μL of the resulting suspension were purified using an Acclaim PepMap 100 column (C18, 100 μM × 2 cm) prior to delivery to an analytical column (Eksigen C18-CL NanoLC column, 3 μm; 75 μm × 15 cm) equilibrated in 5% acetonitrile/0.1% formic acid. Elution was carried out with a linear gradient of 5–35% buffer B in buffer A for 30 min (buffer A, 0.1% FA; buffer B, acetonitrile, 0.1% FA) at a flow rate of 300 nL/min. Peptides were analyzed using an LTQ OrbiTrap Velos Pro (Thermo Scientific) operating in the information-dependent acquisition mode using a top 15 method. MS spectra were acquired in the OrbiTrap analyzer with a mass range of 335–1800 *m*/*z*, with a resolution of 60,000 in the OrbiTrap. Collision-induced dissociation (CID) peptide fragments were acquired in the ion trap with a collision energy of 35, activation energy of 0.25, and 10 ms activation time, with a default charge state of 2 for fragment ions.

Following mass spectrometry, the files containing the peak spectra data were loaded onto Agilent Qualitative analysis software (Agilent Technologies LDA UK Limited, UK). Each file had compounds found by molecular feature and were saved to MGF. OrbiTrap Velos RAW data files were extracted and converted to Mascot generic files (.mgf). All MS/MS samples were analyzed using Mascot (Matrix Science, London, UK; version 2.4.1). Mascot was set up to search the adult *C. daubneyi* transcriptome database^14^ (version 1.0, 73,792 sequence entries; see Bioinformatic Identification of Potential Detoxome Components) assuming the digestion enzyme strict trypsin with one missed cleavage allowed. The transcript data can be accessed from DDBJ/EMBL/GenBank under accession GFUT00000000. Mascot searches were performed using a fragment ion mass tolerance of 0.60 Da and a parent ion tolerance of 10.0 ppm. Carbamidomethylation of cysteine was specified in Mascot as a fixed modification. Gln → pyro-Glu of the N-terminus, deamidation of asparagine and glutamine, oxidation of methionine, dioxidation of methionine, and acetylation of the N-terminus were specified in Mascot as variable modifications.

Scaffold (version Scaffold_4.4.5, Proteome Software Inc., Portland, OR, USA) was used to validate MS/MS-based peptide and protein identifications for EV comparisons. Peptide identifications were accepted if they could be established at greater than 95.0% probability by the Scaffold Local FDR algorithm. Protein identifications were accepted if they could be established at greater than 99.0% probability and contained at least two identified peptides. Protein probabilities were assigned by the Protein Prophet algorithm. Proteins that contained similar peptides and could not be differentiated based on MS/MS analysis alone were grouped to satisfy the principles of parsimony. Proteins sharing significant peptide evidence were grouped into clusters. Additionally, a label-free quantitative analysis was performed in Scaffold for these proteins (15 vs 120 K EVs), with at least two unique peptides, that were present in all three biological replicates. The exponentially modified protein abundance index (emPAI) was used as a quantitative method with a *t* test (Benjamini–Hochberg FDR correction; significance level, *p* < 0.05) as a statistical method. For quantitation, missing values were replaced with a minimum value (default of zero) and normalization was performed with zero as the minimum value.

## Results

### Bioinformatic Discovery of Detoxification Protein Superfamilies

In total, six known detoxification families were analyzed for their presence within the *C. daubneyi* transcriptome. These protein families included three representatives of the key Phase I detoxification enzymes, namely, CYPs, FMOs, and MAOs, and three representatives of key Phase II detoxification enzymes, covering the UGTs, SULTs, and GSTs, all of which have been previously characterized in other Helminth species. Documented proteins confirmed as one of these six protein families were retrieved from the NCBI and Wormbase Parasite databases, including representatives of each class/subfamily if required (see the Supporting Information for full details). Each of the protein sequences retrieved was utilized as queries and subjected to tBLASTn searches against the in-house *C. daubneyi* transcriptome. Transcript sequences with significant similarity (<10^–10^) to query sequences were retrieved and subjected to further characterization.

When examining the Phase I detoxification capacity, transcripts likely representing CYPs and MAOs were revealed, while FMOs were absent, mirroring that observed in the related fluke *F. hepatica* (Table 1). Initially, three transcripts were identified as CYP homologues in *C. daubneyi*. All three transcripts were subsequently subjected to BLAST, Pfam, and Interpro searches, allowing characterization and identification of conserved family-specific domains (Table S1). Each sequence contained either the CYP-specific domain (PF00067) or the oxidoreductase NAD-binding domain (PF00175) with Interpro searches identifying the CYP superfamily domain IPR036396 as well as the CYP conserved site IPR017972. However, only one definitive CYP was identified in the *C. daubneyi* transcriptome (TR23398), with the remaining two sequences (TR22687 and TR23372) demonstrating significant similarity to characterized CYPs containing Interpro domains for oxidoreductase NAD-binding domains, suggesting they are involved in the mechanism or pathway through which CYPs work rather than representing CYPs themselves.

**Table 1:** Number of Transcripts Identified in the Adult *C. daubneyi* Transcriptome of Putative Detoxification Proteins^a^

detoxification enzyme family	no. of hits within C. daubneyi transcriptome	no. of hits within *F. hepatica* genome
cytochrome p450 (CYP)	1	1
flavin-containing monooxygenase (FMO)	0	0
monoamine oxygenase (MAO)	3	3
glutathione transferase (GST)	17	10
UDP-glucurosyltransferase (UGT)	0	0
sulfotransferase (SULT)	11	2

a In total, the transcriptome was mined with model sequences from six detoxification families. Three representative families of Phase I (CYPs, FMOs, and MAOs) are listed in the first three rows, and three representatives of Phase II (GSTs, SULTs, and UGTs) are listed in the last three rows. The number of transcript hits represents the number of sequences confirmed to be representatives of these families. Comparison is made to the related fluke *F. hepatica* based on reports from Cwiklinski et al.^41^ or Stuart et al.^19^ Both species share a similar profile for detoxification, which could support common anthelmintic discovery.

A further five transcripts were identified as having significant similarity to model MAOs in the *C. daubneyi* transcriptome. These five sequences accounted for three proteins, TR24932 (represented by three isoforms), TR22272, and TR16773 (Tables 1 and S1). All of these retrieved transcripts were searched on Pfam for identifying domains relating to MAO activity (PF01593), and of the five sequences, four contained domains specific to MAO activity. However, TR24932 and TR16773 demonstrated significant homology to the lysine-specific histone demethylase, thus leaving only TR22272 as a potential MAO.

When exploring the chosen Phase II detoxification protein Superfamilies, the pattern again mirrored that observed in *F. hepatica,* with representatives of the GSTs and SULTs identified with the absence of UGTs (Table 1). In total, 17 sequences with significant similarity to known model/Helminth SULTs were identified. Following isoform reduction, a total of 11 proteins representing SULTs were identified with a Pfam analysis confirming that these sequences contain one of three sulfotransferase domains, sulfotransferase 1 (PF00685), sulfotransferase 3 (PF13469), and sulfotransferase 4 (PF17784) (Table S1). In addition, BLAST analysis identified 47 additional sequences with significant sequence similarity to GSTs (Table S1). All 47 sequences contained both the N-terminal and C-terminal domains (IPR036282 glutathione S-transferase, C-terminal domain superfamily, and/or IPR004045 glutathione S-transferase, N-terminal).

**Figure 1:**
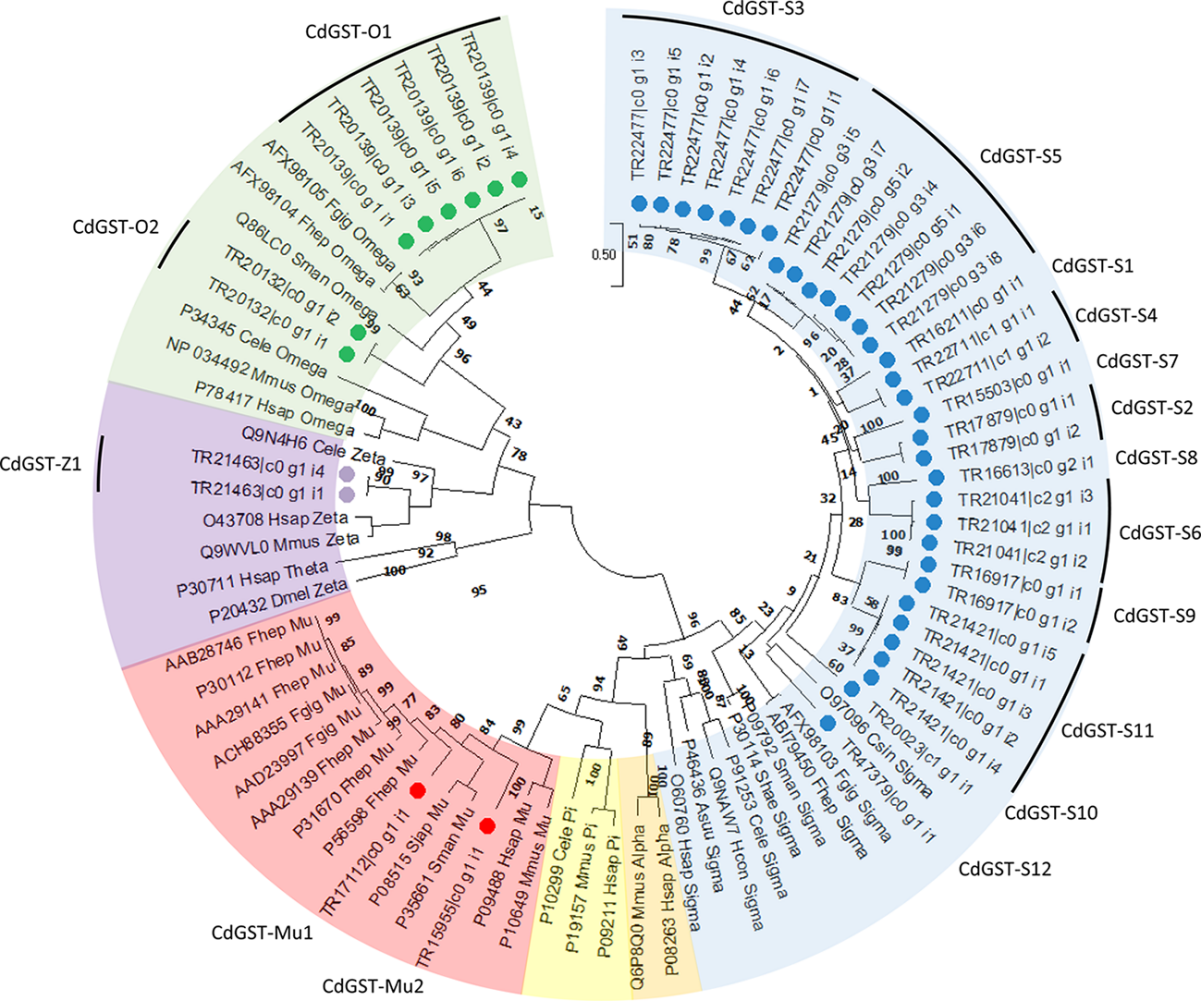
Phylogenetic analysis of the *C. daubneyi* GST superfamily including representative characterized members of seven known classes—Omega, Zeta, Theta, Alpha, Pi, Mu, and Sigma. Constructed using a circular maximum likelihood tree following amino acid alignment on Mega 7.0 with 500 bootstraps and a Poisson correction. Sequence accessions from model organisms on NCBI and Transcript identifiers from transcripts resolved at Aberystwyth University (transcripts are identified with a solid circle). Shading represents the GST class groupings: Sigma, blue; Alpha, orange; Pi, yellow; Mu, red; Zeta/Theta, purple; and Omega, green.

Given the number of classified GST sequences, all were subjected to ClustalW multiple sequence alignment prior to phylogenetic analysis, allowing further elucidation of GST class (Figure 1). Phylogenetic analysis resolved the 17 GST sequences (30 repeat isoforms were removed from the analysis) into one of the four GST classes, namely, Mu, Zeta, Omega, and Sigma-like. In total, 12 sequences were putatively classified as Sigma-like (CdGST-S1–12), with fewer classified as Mu (two sequences; CdGST-Mu1-2), Zeta (one sequence; CdGST-Z1), or Omega (two sequences; CdGST-O1-2) class GSTs. Of note, CdGST-Z1 contained an 82% match to the known Zeta class GST N-terminal motif (**SS**A**S**Y**RVRIAL**; matching residues in bold and underlined) and a 43% match with the known C-terminal motif (**L**NEMD**AF**KK**SHP**DV; matching residues in bold and underlined).

#### *C. daubneyi* GST Proteomic Analysis

**Figure 2:**
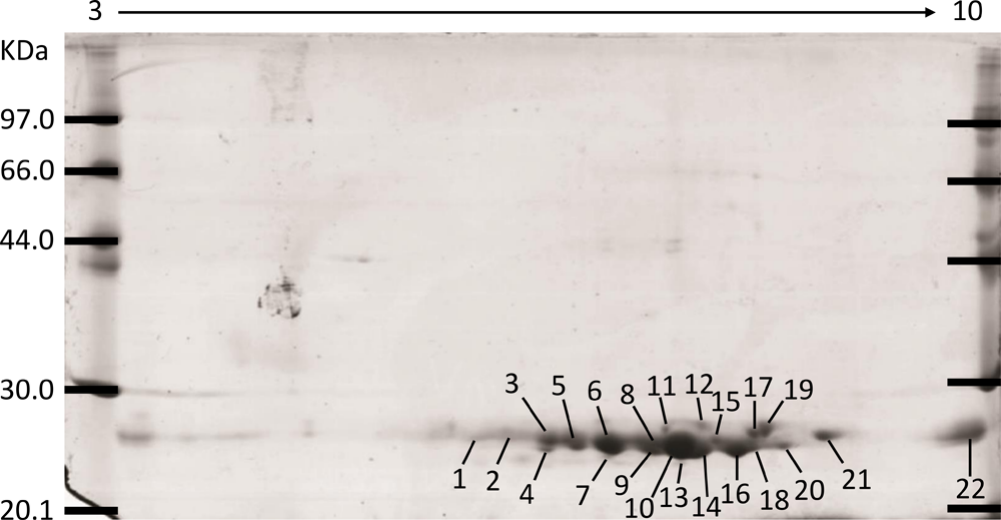
Visualization of high-affinity GSH binding proteins, confirmed as GSTs, of *C. daubneyi* adult worms using two-dimensional gel electrophoresis (2DE): 15 μg of protein was resolved on nonlinear IPG strips separated by charge and in the second dimension by molecular weight on 12.5%, 7 cm polyacrylamide gels (a representative array from *n* = 3 is presented). Numbers indicate putative GST identifications, as found in Table 3.

Bioinformatic interrogation of the transcriptome revealed a dominance of GSTs within the Phase I and II detoxification mechanisms. Given this dominance, a subproteomic analysis of GSH binding proteins (*n* = 3) was conducted to identify GST expression within the somatic proteome of adult worms only (no GST activity could be detected in egg and EV lysates), specifically those with high GSH affinity (Figure 2). Post GSH affinity purification, GSTs represented at least 2.3% of the total soluble cytosolic protein complement of *C. daubneyi* adults (Table 2). Yet, given that a significant proportion of activity was lost (four-fifths of activity, equating to 10673.44 nmol/min) during purification, GSTs may represent up to 10% of the total proteins although this does require confirmation. When this high-affinity GSH binding fraction was analyzed using 2DE, a total of 22 protein spots were observed to be consistent across all three biological replicates. Of these 22 protein spots, all contained GSTs following LC-MS/MS, with 14 spots also containing non-GST components. All GSTs identified belonged to the Mu class or Sigma-like class GSTs. When delineated with the removal of protein isoforms, a total of six distinct GST proteins were identified (Tables 3 and S2), of which five spots were recognized to contain all six identified GSTs (Spots 1, 2, 7, 9, and 14). The GST isotypes represented were two Mu class GSTs (CdGST-Mu1 [TR17112] and CdGST-Mu2 [TR15955]) and four Sigma class representatives (CdGST-S1 [TR16211], CdGST-S3 [TR22477], CdGST-S4 [TR22711], and CdGST-S5 [TR21279]). Specifically, CdGST-S5 and CdGST-Mu1 were the most represented and identified in the 21 protein spots. All GSTs identified contained unique peptides, confirming their presence in each spot (Tables S2 and S3).

**Table 2:** Total and Specific GST Activity Pre and Post Purification for *C. daubneyi* Soluble Somatic Fraction Using the Model Substrate CDNB (*n* = 3)^a^

sample	total activity (nmol/min)	total protein (mg)	specific activity (mean S.D) (nmol/min/mg)
somatic (prepurification)	13161.46	9.1	1367.04 ± 112.10
somatic (postpurification)	2488.02	0.21	2764.47 ± 226.70

a Protein concentrations were determined using the method of Bradford, allowing calculation of the protein recovery rate.

**Table 3:**
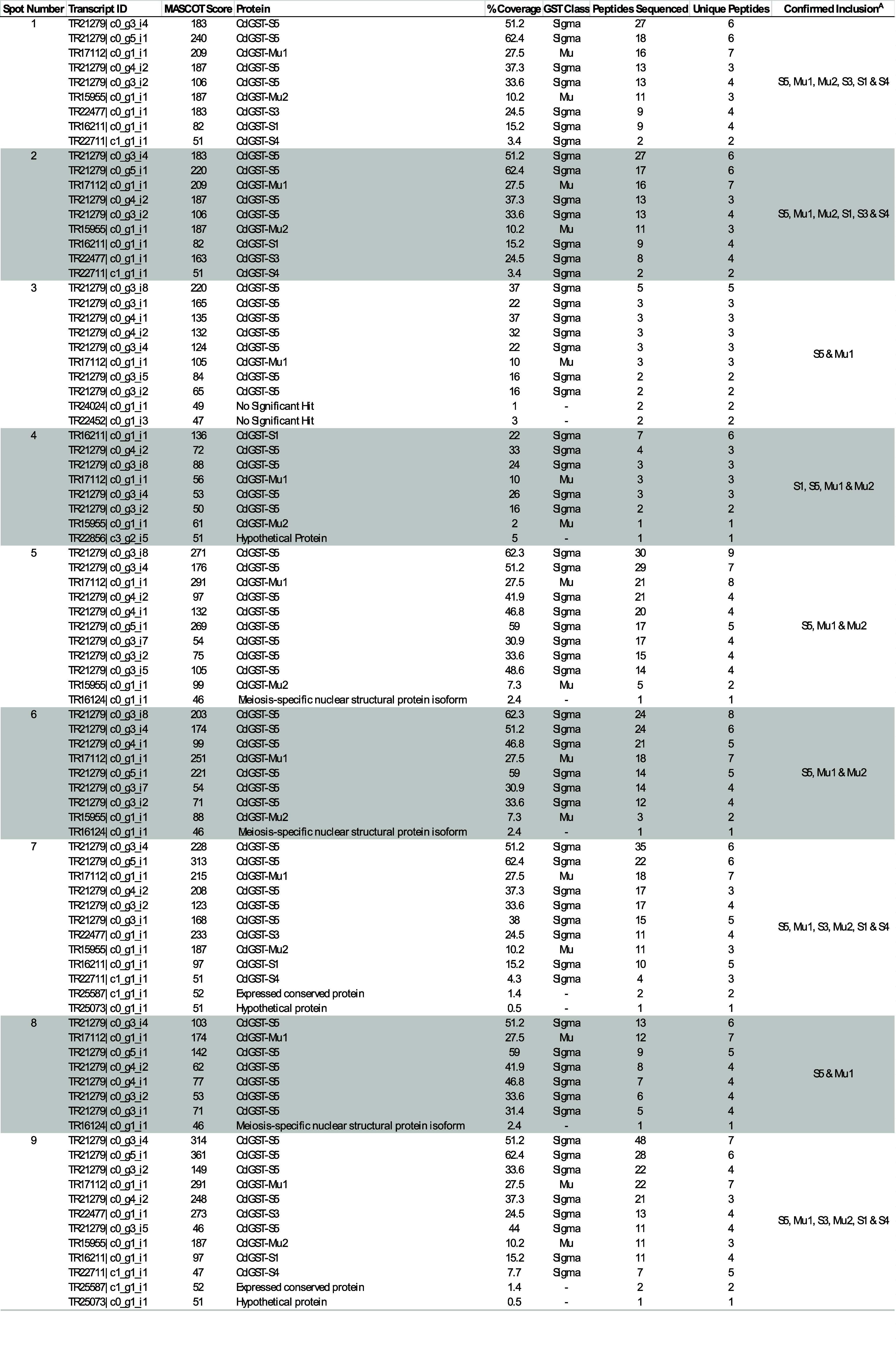

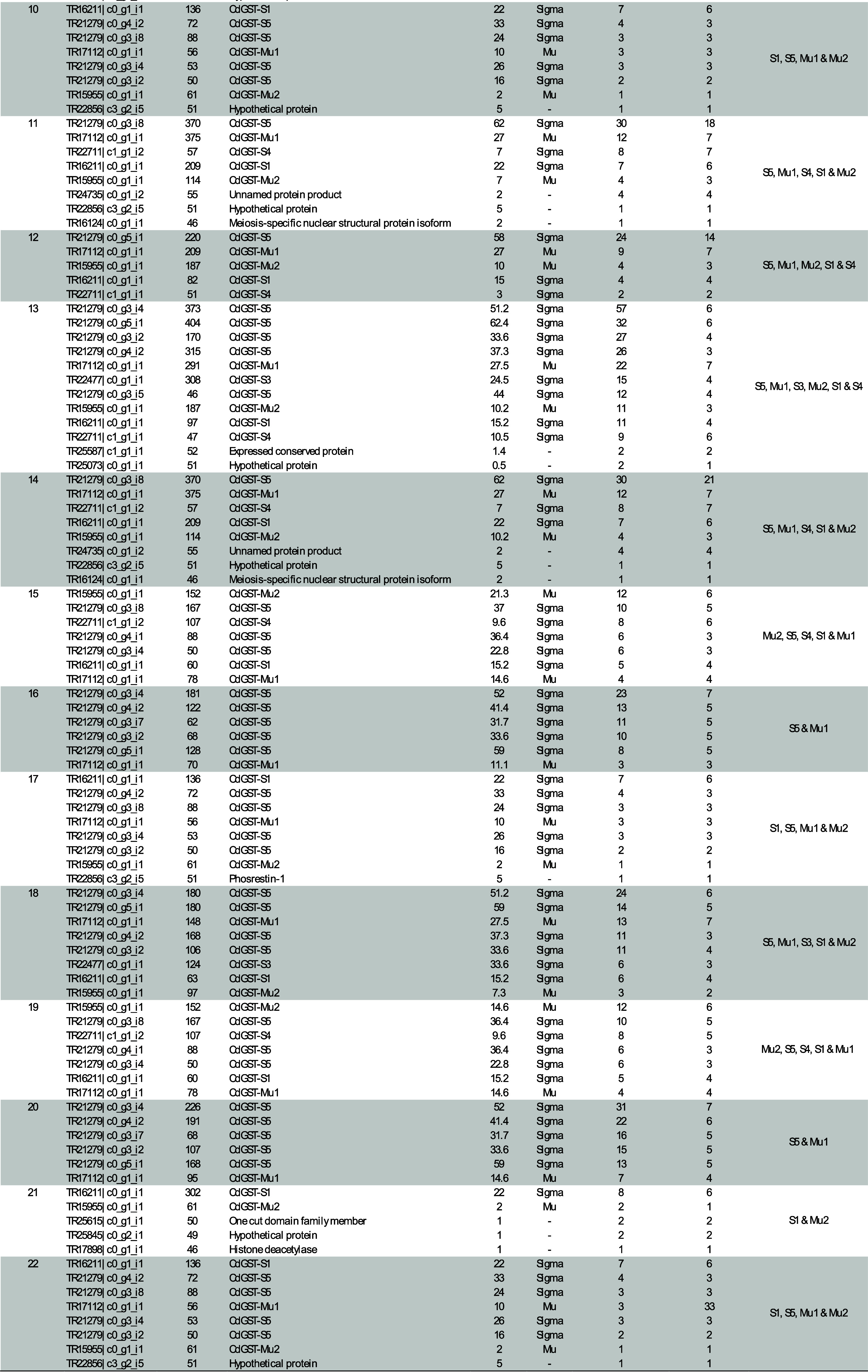
High-Affinity GSH Binding GST Identification^a^

a Proteins identified following 2DE of GSH-purified soluble somatic GST (*n* = 3). Each sequence returned was subjected to BLAST and Pfam analyses. Only sequences above the significance value of 47 were documented. Superscript A: Unique peptides were identified for each GST, with GSTs ordered by abundance based on peptide counts.

To further resolve the GST complement in order to support the identification of low-affinity GSH binding GSTs, adult somatic proteins and a soluble egg extract were subjected to global GeLC proteomic analysis. Adult and egg protein replicates produced consistent and reproducible profiles when they were run on 1D 12.5% polyacrylamide gels (Figure S1). The soluble somatic adult proteome returned a total of 658 protein hits, consistent across all three replicates above the MASCOT significance threshold (Table S4). These included a total of 19 GSTs likely representing 10 distinct enzymes. Of these 10, two proteins were identified as Omega class GSTs (CdGST-O1 [TR20132] and CdGST-O2 [TR20139]). Two proteins identified as Mu class GSTs (CdGST-Mu1 [TR17112] and CdGST-Mu2 [TR15955]), with an additional GST class identified representing a member of the Zeta class GSTs (CdGST-Z1 [TR21463]). The remaining GST protein hits aligned with members of the Sigma class, giving five distinct protein hits (CdGST-S1 [TR16211], CdGST-S2 [TR17879], CdGST-S3 [TR22477], CdGST-S4 [TR22711], and CdGST-S5 [TR21279]).

When exploring the *C. daubneyi* egg proteome, fewer proteins were identified consistently across all triplicate replicates, with only 307 proteins identified (Table S5) despite identifying over 540 proteins in each replicate (Replicate 1: 626 proteins; Replicate 2: 544 proteins; and Replicate 3: 578 proteins). Mining the egg proteome revealed the presence of four GST proteins. Within the egg, and unlike the adult soma, only Mu and Sigma class GSTs were identified (represented by two members of each class). Both previously identified Mu class members, CdGST-Mu1 (TR17112) and CdGST-Mu2 (TR15955), were present, while the Sigma class members consisted of CdGST-S1 (TR16211) and CdGST-S6 (TR21041); the latter was a member not identified within the adult somatic proteome.

Finally, to assess the GST complement of *C. daubneyi* EVs, an additional GeLC approach was performed using biological triplicates of both the 15 and 120 K EV pellets. For the EV analysis, only proteins with two or more unique peptides were accepted, which yielded a total of 1248 protein identifications. After applying a further acceptance criterion (protein presence in at least two of the three biological replicates), the GST EV profile (GSTs identified within the total proteome) almost mirrored that of the GSTs identified within the adult somatic proteome, with no significant differences between the 15 and 120 K EVs, with the identification of CdGST-O1 and O2, CdGST-Mu1 and Mu2, CdGST-Z1 and CdGST-S1, and S3 and S5 with the notable absence of CdGST-S2 and S4. In addition, CdGST-S6 was also absent from the EVs, making this unique to the soluble egg extract. Thus, only three GSTs were consistent across each of the three proteomes examined: namely, CdGST-Mu1, CdGST-Mu2, and CdGST-S1.

**Figure 3:**
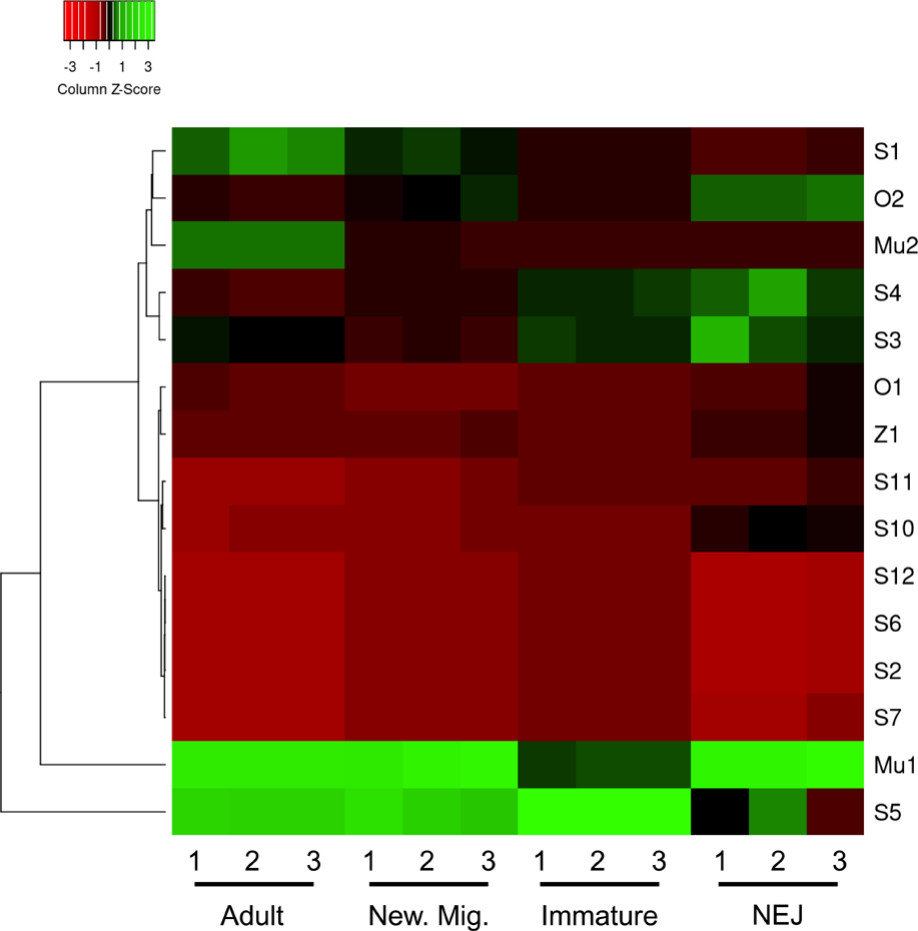
Differential expression of *C. daubneyi* GST transcripts. Heatmap of TPM values scaled by transcript values (where red indicates a low value and green indicates a high value) for each GST identified within the Huson et al.^15^ transcriptome. GSTs were identified through BLAST and compiled using Heatmapper.^42^ New. Mig.: newly migrated; NEJ: newly excysted juvenile.

To assess how their expression changes with rumen fluke development, the TPM values for the identified GSTs were compared across four intramammalian life-cycle stages as previously described (Huson et al.) (Figure 3). Expression data could be retrieved for all identified GSTs except for two Sigma class GSTs, namely, S8 and S9. Most striking was the dominance of CdGST-Mu1 and CdGST-S5 across most life stages examined. Two Sigma class GSTs, CdGST-S3 and -S4, were expressed at a greater level within NEJs and immature migrating fluke, while conversely, CdGST-S1 and CdGST-Mu2 were of a higher expression in adults. Both CdGST-Z1 and CdGST-O1 were expressed at relatively low, yet constitutive, levels across all four life stages. The second Omega class GST, CdGST-O2, was noted to be expressed at its greatest within the NEJ.

### Additional Detoxification Proteins within *C. daubneyi* Global Proteomes

With each of the global adult, egg, and EV (both 15 and 120 K) proteomes resolved, each was examined for the additional detoxification proteins identified through transcriptomic mining. Across all three profiles, no MAOs or CYPs were identified. However, a solitary SULT was identified within the *C. daubneyi* EVs. Specifically, TR19618 was identified in both the 15 and 120 K EV pellets but with a 2.8-fold increase in the 120 K EVs (*P* = 0.0055). Finally, we examined the EV data to explore any likely Phase III detoxification proteins. In both EV pellets, fatty acid binding proteins (FABPs) were identified with one CdFABP III present (TR17138) and two members of the CaFABP IL2 group (TR18162 and TR17688). A fold change was noted for CdFABP III, with a 0.2-fold decrease observed in EVs from the 120 K pellet when compared to those from the 15 K pellet. Additional examination revealed seven ABC family transporters (Table S6) and three acyltransferases (nose resistant to fluoxetine-6 proteins) that have been implicated in drug resistance as cargo components of the EVs. Interestingly, most of the drug transporters were observed at higher levels in the 120 K EVs, of which five (TR25800, TR22250, TR25595, TR25935, and TR21392) were deemed to be statistically significant (*p* < 0.05).

### *Calicophoron daubneyi* EVs Contain Cargo Molecules with Putative Roles in Host/Microbiome Interactions

Given the identification of detoxification proteins within the EVs of *C. daubneyi*, we further expanded this analysis to include EV cargo proteins potentially involved in a wider defense, including host and/or microbiome interactions, and thus the identified proteins were grouped according to their functional annotation. A number of proteins with possible roles in defense against host immunity were also identified and included antioxidants (e.g., thioredoxin, thioredoxin peroxidase) and putative cell adhesion molecules/fusogens (Table S6). Broadly, the abundance of these groups of proteins was similar across both EV subtypes except for a T-cell immunomodulatory protein (TR21689) and a CD59 member (TR18659), both of which were enriched in the 120 K EVs. Two proteins, a saposin (TR18011) and an NK-lysin-like molecule (TR15397), with predicted direct antimicrobial activity were identified in the *C. daubneyi* EVs as well as several proteins with putative roles in host/microbe recognition such as DC-STAMP domain-containing protein 2, basigin (bsg), and lectins. Three proteins with putative roles in cell adhesion (neurotactin, cadherin, and thrombospondin-2) and putative fusogens (rolling stone, dyferlin, myoferlin) were also identified. Most of these proteins had comparable abundance across both EV subtypes except for the lectins, which were expressed at higher levels in the 120 K EVs (Table S6).

### Differing Modes of Biogenesis of the *C. daubneyi* EV Subtypes

With the completed proteomes for both 15 and 120 K EV preparations, we explored the proteins likely involved in EV biogenesis. With this in mind, numerous EV biogenesis proteins, including those with roles in trafficking and release, were identified in the EV total proteome data set. These proteins included 15 members of the endosomal sorting complexes required for the transport (ESCRT) pathway, such as charged multivesicular body proteins 1–5, STAM-binding proteins, Alix, and VTA1. All ESCRT proteins were enriched (eight significantly; *p* < 0.05) in the 120 K EVs, based on triplicate emPAI values (Table S7). In contrast, proteins involved in plasma membrane remodeling, indicative of bleb/microvesicle formation, including ezrin, radixin, and moesin (ERM) and flippase, were significantly enriched (*p* < 0.05) in the 15 K EVs (Table S7). These results indicate that 120 K EVs are derived from the endosomal compartment, whereas 15 K EVs may originate from the plasma membrane. Numerous other proteins involved in vesicle trafficking, such as Rabs, GTPases, SNAREs, and vesicle membrane proteins (e.g., tetraspanins), were also identified in both EV subtypes, with most enrichment (*p* < 0.05) occurring in the 120 K EVs.

## Discussion

Currently, in the absence of vaccines, chemotherapeutic control, with anthelmintics, remains the main treatment strategy utilized in both the control of symptoms and the elimination of Helminth infections in humans and animals.^43^ Thus, anthelmintics have been intensively used for livestock production worldwide, which has led to the development of resistance to all those licensed for use.^44^ Owing to the lack of development of new treatment options, understanding the mechanisms through which helminths develop resistance is crucial, allowing the adaptation of current treatments to prevent resistance and surveillance of its development while also allowing the elucidation of novel treatment targets.^45^ It is well recognized that drug resistance can be facilitated by the mode of action of XMEs, and bioinformatics has revealed predicted drug metabolism capacities and potential drug resistance mechanisms in different parasites.^46^*Calicophoron daubneyi* is the causative agent of a newly emerging disease with limited anthelmintic options. Thus, it is imperative to understand how this parasite could metabolize anthelmintics to support drug development and reveal potential resistance routes. We have discovered the *C. daubneyi* detoxome capacity through a combined bioinformatic and proteomics lead approach.

Of the six known detoxification families examined in silico, two were absent from the *C. daubneyi* transcriptome, specifically, Phase I FMOs and the Phase II UDP-glucuronosyltransferases (UGTs). Genome-level studies in related helminths have noted the absence of FMOs in an array of parasitic flatworms^47^ despite the detection of monooxygenase activity using methimazole indirectly assuming FMO activity in *F. hepatica*.^3^ Thus, the absence of FMOs at the genome level is not unexpected and may reflect their residence in an anaerobic environment. With regard to helminths, UGTs have thus far only been identified in nematode species, such as the free-living model species *C. elegans* and the parasitic *Hemonchus contortus*.^48^ The failure to detect UGTs in this parasitic flatworm investigation is therefore in concordance with related trematode helminths and is thus unsurprising in their absence from the *C. daubneyi* detoxome.

Of the hundreds of CYPs characterized across taxa, only a low percentage has been identified as playing a role in xenobiotic detoxification^49^; for example, approximately 21% of the human CYPs are involved.^50^ Reductionist biochemical studies failed to detect CYP detoxification enzymatic activity in parasitic helminths,^16^ but later genome sequencing has predicted CYPs from numerous platyhelminth species. A single CYP has been identified in *F. hepatica*,^41^*Schistosoma japonicum*, *Schistosoma mansoni*, *Schistosoma hematobium,* and *Opisthorchis felinus,*(51) and for the first time, a single CYP has been identified in *C. daubneyi*. Given that P450s require oxygen for function, a reduction in CYP complexity could represent a bespoke housekeeping role, including a defined detoxification mechanism in anaerobic environments.^47^ For example, *S. mansoni* CYP is predicted to have a crucial role in egg development, and in *H. contortus*, it plays a major role in larval stage development.^52^ Given CYPs are membrane-bound proteins, the absence of CYPs in the soluble somatic and egg proteomes is not surprising, yet the EV proteomes would have likely identified them if present. Thus, the absence in the EV likely further supports a housekeeping role in development rather than detoxification. However, further investigation into CYPs in adult, juvenile, and eggs of *C. daubneyi* within membrane fractions could be of significant interest.

An examination into the involvement of Phase I MAOs in xenobiotic detoxification has been largely neglected, mainly due to interest in the research of the CYPs.^53^ MAOs are capable of metabolizing amine-containing drugs and are characterized as a family of enzymes able to catalyze the biotransformation of xenobiotics leading to their excretion from mammals.^54^ Only two MAOs have been characterized in humans, namely, MAO-A and MAO-B, each of which has been identified as having vastly different substrate specificity.^55^ Within the current bioinformatics investigation, three predicted *C. daubneyi* proteins were identified that contained the amino oxidase Pfam domain (PF01593), although two are likely lysine-specific histone demethylases, leaving one recognized MAO. Of note, neither the defined MAO nor the two Lysine-specific histone demethylases were identified within the proteomes examined and, as with CYPs, likely reflect their expression in membrane fractions (likely mitochondrial) and thus absent from soluble proteomes.

The present study also identified 11 proteins with Pfam identifiers for Phase II SULT activity: namely, sulfotransferase 1 (PF00685), sulfotransferase 3 (PF13469), and sulfotransferase 4 (PF17784). The full mechanism of SULT activity during detoxification is still not fully resolved, yet it is proposed to function through neutrophilic attack of the sulfur hydroxyl group on 5′-phosphoadenosine-3′-phosphosufate (PAPS), resulting in its transfer to the substrate.^56^ Previous research into Helminth species indicated their inability to carry out sulfate activation and predicted the absence of sulfotransferase activity.^57^ However, *S. mansoni* is now known to contain a sulfotransferase gene as the activating enzyme for oxamniquine (OXA). Resistance to OXA through sulfotransferase activity has also been demonstrated through crystallography of its interaction with OXA, showing its ability to transfer sulfate groups from PAPS to OXA,^58,59^ as well as confirmation of OXA resistance following RNAi knockdown of Schistosome SULT Smp_089320.^59^ This is the first report of SULTs in *C. daubneyi* and, given a novel role in the development of anthelmintic resistance in other helminths, could prove useful in the future design of drug compounds to tackle *C. daubneyi* infections. This is especially so with the identification of a SULT enriched within the 120 K EVs, which may be released from the tegumental surface,^23^ a key site of anthelmintic action in trematodes, as demonstrated in the related *F. hepatica*.^23,60^ Given the broad similarity across Phase I and II detoxification between liver and rumen flukes (Table 1), supporting common anthelmintic discovery programs, this increase in SULTs represents an increased capacity within *C. daubneyi,* which may present future challenges to discovery programs utilizing compounds potentially metabolized via SULTs.

By far, the largest Phase I or II detoxification family identified within *C. daubneyi* was that of the Phase II GST family. GSTs are presumed to be of much importance within platyhelminths, including *Clonorchis sinensis* and *Fasciola* species, given that they account for approximately 4% of the adult soluble protein fraction in the closely related trematode *F. hepatica.* Within *C. daubneyi*, GSTs accounted for a comparable 2.3% of the adult soluble protein fraction, thus supporting their importance in platyhelminth species, although GSTs may represent up to 10% of the total proteins. In *F. hepatica*, GSTs are also hypothesized to be involved in the development of anthelmintic resistance,^19,61^ with four classes of GST resolved using multiomics approaches, specifically Omega (ω), Mu (μ), Sigma (σ), and Zeta (ζ).^7,9^ This profile containing four classes of GSTs was mirrored within *C. daubneyi*. In comparison to *F. hepatica*(19) and *F. gigantica*,^9^ and following phylogenetic analysis, *C. daubneyi* contained an identical number of Omega and Zeta class GSTs at two and one GSTs, respectively. However, there is a reduction in the number of Mu class isoforms, from five in fasciolids to two in *C. daubneyi*, with an increase in Sigma class GSTs: 12 Sigma-like GSTs in *C. daubneyi* and only two within fasciolids. The expansion of Sigma-like class GSTs observed in *C. daubneyi* has also been identified in helminths previously, with studies of *O. viverrini* and *C. sinensis* also observing an increased number of Sigma/Sigma-like GSTs present: five identified in both species.^62^ Expansion within *O. viverrini* and *C. sinensis* has been attributed to the parasite’s migration through the host to reach its definitive residency and Sigma class GST's involvement in parasite migration. Thus, it is likely that the Sigma class GST expansion in the rumen fluke may reflect residency within the rumen, following a period of migration along the gastrointestinal tract. Huson et al.^15^ identified a number of GSTs in the secretome: in both adult and newly excysted juvenile (NEJ) secretomes, six GST members, representing Mu class (CdGST-Mu1 [Cdaub_09334] and CdGST-Mu2 [Cdaub_13573]), Omega class (CdGST-O1 [Cdaub_09339] and CdGST-O2 [Cdaub_03669]), and Sigma class (CdGST-S3 [Cdaub_16419] and CdGST-S5 [Cdaub_02527]), were identified. However, in the migrating juveniles, an additional two Sigma class isoforms (CdGST-S4 [Cdaub_17204] and CdGST-S11 [Cdaub_08381]) were also identified, supporting the role of expanded Sigma class GSTs during the migration along the gastrointestinal tract. Of note, CdGST-S4 was one of the most abundant GSTs within the NEJ.

When investigating the high GSH affinity fraction of *C. daubneyi* GSTs, via a subproteomic approach, both Mu class GSTs, CdGST-Mu1 and CdGST-Mu2, were purified and identified. However, only four of the five previously identified Sigma-like class GSTs were observed within this fraction, likely reflecting their expression levels in adults; CdGST-Mu1, -Mu2, -S1, and -S5 are the most highly expressed GSTs in adults, suggesting that CdGST-S2 is the only low GSH affinity Sigma class GST. Low and high GSH affinity GSTs from Mu and Sigma classes have been demonstrated previously in *F. hepatica,*(19) and thus, these low-affinity CdGSTs may represent GSTs tightly bound to a “blocking” factor, as proposed by Brophy et al.,^63^ or are simply low-affinity isoforms as observed for FhGST-Mu5.^19^ As expected, none of the recognized low GSH binding affinity GSTs were purified and analyzed on 2DE arrays with the absence of CdGST-O1, -O2, and -Z1 as demonstrated for *F. hepatica*(7) and *F. gigantica*.^9^

However, CdGST-O1, -O2, and -Z1 were identified in global-proteomic profiles when examining the adult somatic and EV profiles. Omega class GSTs, from the related *F. hepatica*, have demonstrated thiol transferase activity.^64^ Therefore, a likely supporting role in detoxification is identified for Omega class GSTs through the repair of oxidation damage following protein S-thiolation, potentially from GSTs protein–S-S-glutathione, through dethiolation.^65^ Thus, Omega class GST identification as part of the detoxome in an adult rumen fluke is logical. Zeta class GSTs are known to exert peroxidase activity^66^ and therefore will likely perform key roles as part of the general detoxome. However, peroxidase activity in Helminth Zeta class GSTs is yet to be confirmed, and so it is likely that CdGST-Z1 will primarily function as a maleylacetoacetate isomerase involved in the metabolic degradation of phenylalanine and tyrosine.^66^

Interestingly, neither Omega nor Zeta class GSTs were observed in eggs despite the identification of Omega class GSTs in the eggs of *F. hepatica*.^35^ In general, the expression of GSTs within the eggs was significantly reduced with only the two Mu class representatives (CdGST-Mu1 and -Mu2) and two Sigma class representatives (CdGST-S1 and -S6), with S6 being novel to the egg proteome. Given the reduction of GSTs in the egg, it is likely that a full detoxification capacity is not required, and so alternative functions may need to be assigned to the identified GSTs. Specifically, CdGST-S6, the egg-specific isoform, may be of more relevance for a potential prostaglandin D2 (PGD2) synthase activity, as shown for other helminths,^17,67^ and thus function in egg development and embryogenesis. However, PGD2 activity will need confirmation in CdGST-S6, and indeed other Sigma class GSTs, especially given that the closest rumen fluke homologue of FhGST-S1, the *F. hepatica* Sigma class GST with proven PGD2 synthase activity, is CdGST-S11 (enriched in migrating NEJs) and not the egg-specific CdGST-S6.

Both 15 and 120 K EVs were also explored for their GST complement and revealed a profile that almost mirrored that of the adult somatic proteome, thus demonstrating the expansion of the detoxome into the EVs. Both Omega class, both Mu class, the Zeta class, and five of the Sigma class GSTs were revealed, in agreement with that observed for liver fluke adult EVs as cargo and surface-exposed^18,22^ in addition to Sigma class identification in miracidial EVs.^68^ The notable absences in both rumen fluke EV profiles were that of CdGST-S2 and -S4. None of the GSTs were significantly expressed in greater quantities in either EV subpopulation, suggesting the importance of detoxification in both subpopulations derived from the intestinal lumen or the tegumental surface and protonephridial system.^23^ Unfortunately, experimental GST purification of lysed *C. daubneyi* EVs did not return a significant concentration of protein to confirm high-affinity EV-specific GSTs.

The detoxome observed within EV populations extended to the identification of a SULT that was observed in both subpopulations but enriched in the 120 K EVs. SULTs are known components of many mammalian EVs and have been located in rat hepatocyte EVs.^69^ Given SULTs are key components of Phase II detoxification, it is highly likely that the identified SULT will be adding to the detox capacity of EVs. The enrichment within 120 000 EVs may suggest a focus of this EV subpopulation in xenobiotic detox associated with the complex rumen environment.

The *C. daubneyi* EVs also contained Phase III detoxification components, including FABPs, proposed toxin binding proteins, and ABC transporters. Within the EVs, one CdFABP III was identified and two CdFABP IL2 members, with the former increased in 15 K EVs. FABPs are suggested to act as sequesters of xenobiotics for removal in fasciolids,^70^ with Esteves and Ehrlich^71^ proposing that *Schistosoma japonicum* FABP will also act as a drug sequester. Thus, the presence of FABPs in EVs further supports the EV detoxification capacity. Alternatively, with an increase of CdFABP III in 15 K EVs, EVs likely derived from the intestinal surface,^23^ FABP as EV cargo will act as sequesters of fatty acids from digesta. The 120 K EVs were further enriched with ABC transporters, suggesting a more significant role for the tegument and protonephridial-derived EVs in detoxification. EVs originating from the endosomal pathway, such as the 120 K EVs in related flukes,^23^ have been demonstrated in cancer cells to be key to drug uptake and removal, thus buffering the impact of chemotherapeutics.

The depth of proteomic characterization of the *C. daubneyi* EVs conducted here presented an opportunity to examine the potential role of 15 and 120 K EVs to elicit an effect on the host. The recent discovery of FhGST-O2 modulating the physiological functions of macrophages^64^ suggests *C. daubneyi* EVs, containing two Omega class GSTs, may also be involved in immunomodulatory and anti-inflammatory roles during the infection. Furthermore, previous research into *C. sinensis* has highlighted the specific EV packaging of a Sigma class GST allowing the activation of M2 macrophages facilitating Helminth defense.^62^ However, the closest homologue in *C. daubneyi* to that of the well-documented immune modulating FhGST-S1,^17^ namely, CdGST-S11, was absent in any proteomic analysis and expressed at very low levels across ontogenic stages.

EV biogenesis in helminths is not yet fully understood, with most evidence for release mechanisms coming from proteomic analysis of isolated vesicles, immunolocalization using EV markers, ultrastructural studies, or the use of chemical inhibitors of EV formation.^22,23,36,60,72^ Nevertheless, the enrichment of ESCRT components in the *C. daubneyi* 120 K EV pellet would suggest that these vesicles are derived from the endosomal system, while the association of molecules involved in plasma membrane remodeling with the 15 K EVs is indicative of microvesicle/bleb formation. A similar differential distribution of EV biogenesis proteins has been observed in *F. hepatica* EVs.^22,60^ Thus, it appears that *C. daubneyi* also secretes different EV subpopulations with distinct cargo and mechanisms of biogenesis, with yet-undetermined functionality. It is noteworthy, however, that the *C. daubneyi* EVs contained proteins with putative roles in wider defense mechanisms beyond detoxification of xenobiotic compounds. These included the antioxidant thioredoxin peroxidase that may protect the parasite against reactive oxygen species but can also modulate host macrophage phenotype and function,^73^ a homologue of a T-cell immunomodulatory protein from the cestode *Echinococcus multilocularis*,^74^ a CD59 family member which regulates complement-mediated cell lysis,^75^ and the transmembrane protein 106A, an important regulator of macrophage homeostasis.^76^

In addition to immunomodulators, molecules with the potential to interact with rumen microbiome components were also identified within *C. daubneyi* EVs. These included a number of lectins that can function as pattern recognition receptors^77^ as well as saposin and an NK-lysin homologue, which display direct antimicrobial activity via the lysis of bacterial cell membranes.^78^ This is intriguing given the recent discovery that *C. daubneyi* EVs can modulate rumen microbiome population diversity via antimicrobial activity.^20^ Rumen fluke–microbiome interactions are a currently understudied axis of infection but one that will be key to understanding the impact of paramphistomosis on production parameters such as feed conversion efficiency and greenhouse gas emissions from livestock^79^ as we work toward net zero carbon emission targets.

The current work provides the most extensive coverage of the *C. daubneyi* detoxification potential to date. This detox capacity also likely extends into the EVs, specifically those EVs originating through the endosomal pathway. With this recognition, new opportunities arise to investigate drug efficacy and potential mechanisms of resistance. This is in addition to the extent to which EVs may exert their own detoxification capacity within the context of the parasite and host interaction given it may be likely that EVs play at least a supportive role in removing and metabolizing xenobiotics from the immediate environment.^80^

## Data Availability

Proteomics data from LC-MSMS analysis has been deposited to the ProteomeXchange Consortium via the PRIDE partner repository: Proteome data set identifier PXD014550.

## Supporting Information Available

List S1, sequences utilized within the bioinformatics searches for Phase I and II detoxification families within the *C. daubneyi* transcriptome; and Figure S1, representative 1D 7 cm protein profiles of adult *C. daubneyi* soluble somatic and soluble egg extract fractions as part of a global GeLC proteomic approach (PDF)
Table S1, detoxification capacity bioinformatics BLAST output from searching six known detoxification families from Phase I and Phase II identified in the *C. daubneyi* transcriptome; Table S2, full summary of the proteins identified in the 2DE GST proteome analysis from *C. daubneyi*; Table S3, *C. daubneyi* GST unique peptides; Table S4, summary of the proteins identified in the soluble somatic proteome of adult *C. daubneyi*; Table S5, summary of the proteins identified in the egg proteome of adult *C. daubneyi*; Table S6, defense proteins identified in the 15 and 120 K proteome of adult *C. daubneyi* 15 and 120 K EVs; and Table S7, biogenesis proteins identified in the 15 and 120 K proteome of adult *C. daubneyi* 15 and 120 K EVs (XLSX)
