# Supplementary material for: Detoxome Capacity of the Adult Rumen Fluke Calicophoron daubneyi Extends into Its Secreted Extracellular Vesicles
Source: J Proteome Res. 2025 Jan 20;24(2):624–38. doi: 10.1021/acs.jproteome.4c00615 (PMC11812014; doi:10.1021/acs.jproteome.4c00615)
Supplement: Supplementary file 1 — pr4c00615_si_001.pdf [file pr4c00615_si_001.pdf]

SUPPORTING INFORMATION:

The detoxome capacity of the adult rumen fluke *Calicophoron daubneyi* extends into its secreted extracellular vesicles

Allen, Nathan Rhys[1], Huson, Kathryn M.[2], Prchal, Lukas[1], Robinson, Mark W.[2], Brophy, Peter M.[1] & Morphew, Russell M.[1*]

[1]Department of Life Sciences, Aberystwyth University, Aberystwyth, Wales, UK. SY23 3DA.

[2]School of Biological Sciences, Queen's University Belfast, Belfast, Northern Ireland, UK. BT9 5BY.

[*]Corresponding author

Corresponding author email: rom@aber.ac.uk

TABLE OF CONTENTS

List S1: Sequences utilised within the bioinformatics searches for Phase I and II detoxification families within the *C. daubneyi* transcriptome.

Figure S1: Representative 1D 7cm protein profiles of adult *C. daubneyi* soluble somatic and soluble egg extract fractions as part of a global GeLC proteomic approach.

Table S1: Detoxification capacity bioinformatics BLAST output from searching 6 known detoxification families from Phase I and Phase II identified in the *C. daubneyi* transcriptome.

Table S2: Full summary of the proteins identified in the 2DE GST proteome analysis from *C. daubneyi*

Table S3: *C. daubneyi* GST unique peptides.

Table S4: Summary of the proteins identified in the soluble somatic proteome of adult *C. daubneyi.*

Table S5: Summary of the proteins identified in the egg proteome of adult *C. daubneyi*.

Table S6: Defence proteins identified in the 15K and 120K proteome of adult *C. daubneyi* 15K and 120K EVs.

Table S7: Biogeneis proteins identified in the 15K and 120K proteome of adult *C. daubneyi* 15K and 120K EVs.

List S1: Sequences utilised within the bioinformatics searches for Phase I and II detoxification families within the *C. daubneyi* transcriptome.

CYPs: *F. hepatica* (THD26616), *C. sinensis* (RJW68120), *O. felineus* (AEI26271), *E. granulosus* (CDJ17920) & *S. mansoni* (ALV83720).

MAOs: *S. mansoni* (CCD80017.1), *S. haematobium* (XP_012800160.1), *O. viverini* (XP_009164023.1), *C. sinensis* (GAA53069.1), *C. sinensis* (GAA28252.1), *C. sinensis* (GAA57364.1), *O. viverini* (XP_009172770.1), *S. mansoni* (CCD83018.1) & *O. viverini* (XP_009177175.1).

FMOs: *C. elegans* (CE31710).

GSTs: *F. gigantica* (ACH88355.1, AFX98103.1 & AFX98105.1), *F. hepatica* (AAA29139.1, 1905266C, 1905266A, P31670.3, P56598.2, 1905266D, ABI79450.1 & AFX98104.1), *H. sapiens* (P09488.3, P08263.3, P09211.2, O60760.3, P30711.4, O43708.3 & P78417.2), *M. musculus* (P10649.2, Q6P8Q0, P19157.2, Q9JHF7.3, Q64471.4, Q9WVL0.1 & O09131.2), *S. japonicum* (P08515.3), *S. mansoni* (P35661.2, P09792.1 & Q86LC0), *C. elegans* (P10299.1, P91253.1, Q9N4H6 & P34345.1), *S. haematobium* (P30114.1), *C. sinensis* (O97096), *A. suum* (P46436.3), *H. contortus* (Q9NAW7), *D. melanogaster* (P20432.1), *M. destructor* (ABG56084.1), *L. lineolaris* (ABC46450.1), *B. dorsalis* (AFJ05093.1).

SULTs: *H. sapiens* (AAI10888.1, NP_001045.1, AAH78144.1, NP_808220.1, AF186255_1, O00338.1, AAB82293.1, CAG30474.1, AAC78498.1, CAG28549.1, NP_006579.2, NP_001008743.1 & NP_055280.2), *M. musculus* (AAH24361.1, NP_061221.2) *C. sinensis* (GAA54196.1 & GAA47314.1), *O. viverrini* (OON24041.1, XP_009169455.1 & XP_009170672.1), *S. haematobium* (A0A095A1P1), *S. japonicum* (C1LFD7), *S. mansoni* (XP_018645283 & 4MUB_A), *C. elegans* (O17645 & O77081).

UGTs: *C. sinensis* (GAA43179.2, GAA36922.2 & GAA52572.1), *S. mansoni* (CCD78639.1 & XP_002576438.1), *S. japonicum* (CAX69946.1)

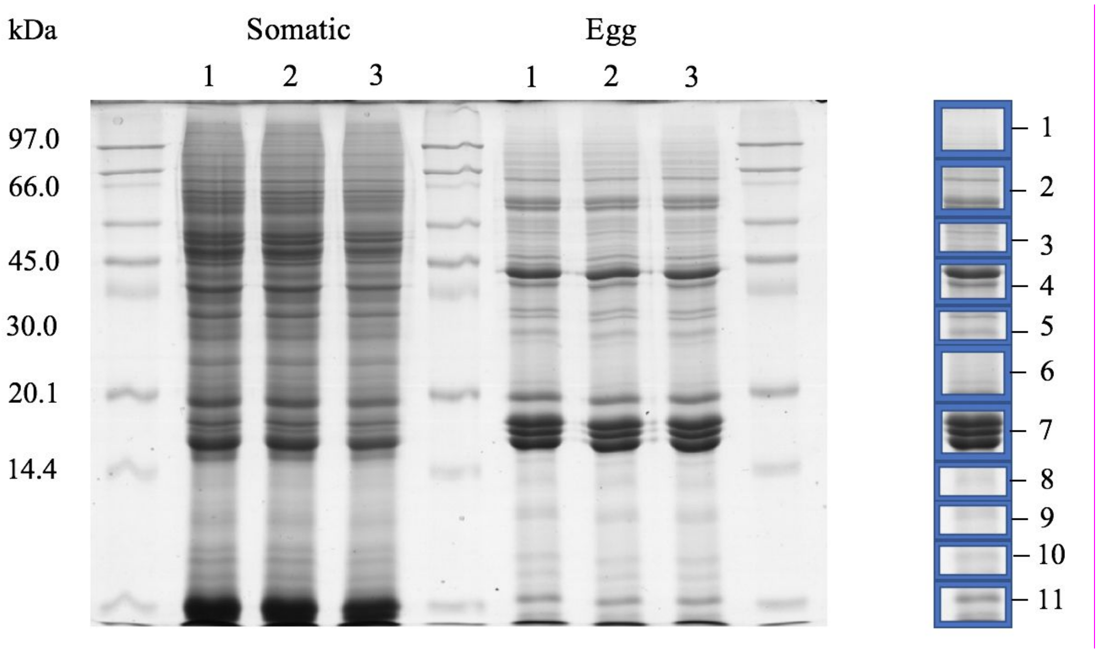

Figure S1: Representative 1D 7cm protein profiles of adult *C. daubneyi* soluble somatic and soluble egg extract fractions as part of a global GeLC proteomic approach. A total of 10 µg of each sample was loaded for each lane and separated by 12.5% SDS-PAGE, the gel was then Coomassie Blue stained for visualisation before mass spectrometry (n=3). Representative division of each lane into bands for LC-MS/MS preparation are given on the right.

Table S1: Detoxification capacity bioinformatics BLAST output from searching 6 known detoxification families from Phase I and Phase II identified in the *C. daubneyi* transcriptome. In total 3 Cytochrome P450 (CYP) contigs returned significant similarity to representative sequences (<E^{-10}). Blast identification and top accession hits were recorded as well as Pfam IDs PF00067 (Cytochrome P450 domain) and PF00175 (Oxidoreductase NAD-binding domain). Five Monoamine oxidase (MAOs) transcripts were identified, relating to 3 protein sequences. Resolved sequences were submitted to BLAST and Pfam searches for species identification and Pfam IDs (PF01593 – Amine Oxidase). For sulfotransferases (SULTs) contigs a total of 17 returned significant homology to representative sequences (<E-10). Blast identification and top accession hits were recorded as well as Pfam ideas PF13469, PF17784, PF00685 and PF03567 (Sulfotransferase families) were also identified. Finally, Glutathione transferase (GSTs) contigs totalled 46 with significant homology to representative sequences (<E^{-10}). Blast identification and top accession hits were recorded as well as Pfam IDs – N-terminal (PF02798 and PF13417) and C-terminal (PF00043, PF14497, and PF13410). Isoforms are grouped together for convenience.

Table S2: Full summary of the proteins identified in the 2DE GST proteome analysis from *C. daubneyi*

Table S3: *C. daubneyi* GST unique peptides. All GSTs identified during the 2DE analysis contained unique peptides. GSTs CdGST-Mu1, CdGST-Mu2, CdGST-S1 and CdGST-S4 only contained unique peptides. However, CdGST-S3 and S5 shared 3 peptides between them (Peptides shaded grey).

Table S4: Summary of the proteins identified in the soluble somatic proteome of adult *C. daubneyi.*

Table S5: Summary of the proteins identified in the egg proteome of adult *C. daubneyi*.

Table S6: Defence proteins identified in the 15K and 120K proteome of adult *C. daubneyi* 15K and 120K EVs.

Table S7: Biogeneis proteins identified in the 15K and 120K proteome of adult *C. daubneyi* 15K and 120K EVs.
